# Advances in the Neuro-Rehabilitation of Parkinson’s Disease: Insights from a Personalized Multidisciplinary Innovative Pathway

**DOI:** 10.3390/biomedicines12112426

**Published:** 2024-10-23

**Authors:** Maria Grazia Maggio, Mirjam Bonanno, Alfredo Manuli, Rosaria De Luca, Giuseppe Di Lorenzo, Angelo Quartarone, Rocco Salvatore Calabrò

**Affiliations:** 1 IRCCS Centro Neurolesi Bonino-Pulejo, 98124 Messina, Italy; mariagrazia.maggio@irccsme.it (M.G.M.); rosaria.deluca@irccsme.it (R.D.L.); giuseppe.dilorenzo@irccsme.it (G.D.L.); angelo.quartarone@irccsme.it (A.Q.); roccos.calabro@irccsme.it (R.S.C.); 2 A.O.U. Policlinico “G. Martino”, Via Consolare Valeria, 98124 Messina, Italy; manulialfredo@gmail.com

**Keywords:** cognitive rehabilitation, executive functions, neurorehabilitation, Parkinson disease, robotics, virtual reality

## Abstract

**Background/Objectives**: Parkinson’s disease (PD) is a progressive neurodegenerative disorder that requires comprehensive and personalized rehabilitation. This retrospective study focused primarily on the usability and patient acceptability of the innovative pathway. In addition, the secondary objective was to evaluate the effectiveness of a personalized and multidisciplinary rehabilitation pathway on cognitive function, especially executive functions. **Methods:** We conducted a retrospective study on 80 patients with PD (Hoehn and Yahr scores 1–3). Patients were divided into an experimental group (EG), which received the innovative pathway, and a control group (CG), which received traditional therapy. The rehabilitation program included three phases: initial outpatient assessment, a two-month inpatient program, and a telerehabilitation phase in a day hospital (DH) or home environment. Interventions combined traditional therapies with treatments based on robotic and virtual reality. Cognitive assessments (Mini Mental State Examination—MMSE—and frontal assessment battery—FAB), mood (Hamilton Rating Scale—Depression—HRS-D), anxiety (HRS-Anxiety—HRS-A), and goals achievement (GAS) were the primary outcome measures. **Results**: At baseline, there were no significant differences between the groups in terms of age, gender, education, or test scores. After rehabilitation, EG showed significant improvements in all measures (*p* < 0.001), particularly in cognitive tests and goal achievement. CG improved in GAS (*p* < 0.001) and mood (HRS-D, *p* = 0.0012), but less than EG. No significant changes were observed in the MMSE of CG (*p* = 0.23) or FAB (*p* = 0.003). **Conclusions**: This study highlights the high usability and acceptability of VR and robotics in PD rehabilitation, contributing to improved adherence and patient engagement. The experimental group showed greater cognitive benefits, particularly in executive functions. These results are in line with the existing literature on personalized technology-based rehabilitation strategies for PD.

## 1. Introduction

Parkinson’s disease (PD) is a progressive neurodegenerative disorder that primarily affects the motor system. It is estimated that over 10 million people worldwide live with PD; its incidence increases with age and the disease is more common in men than women [1]. Clinically, PD is characterized by the hallmark motor symptoms of bradykinesia, resting tremor, rigidity, and postural instability, along with non-motor symptoms such as cognitive deficits, depression, anxiety, and sleep disturbances [2,3]. In the early stages, diagnosis is largely clinical, and the progression of motor symptoms is typically assessed using tools such as the Hoehn and Yahr (H&Y) scale [4] and the Unified Parkinson’s Disease Rating Scale (UPDRS) [5]. However, a critical and often underestimated aspect of PD is the decline in cognitive function [6], which can have a profound impact on patients’ quality of life [6,7,8].

From a therapeutic point of view, the treatment of PD consists of a combination of pharmacological, surgical, and rehabilitative approaches. Dopaminergic drugs such as levodopa remain the gold standard for managing motor symptoms, but as the disease progresses, motor complications such as fluctuations and dyskinesias emerge, limiting long-term efficacy [9]. The need for early and multidisciplinary intervention is rooted in the awareness that PD is not simply a motor disorder, but a multifaceted condition that affects multiple functional systems [10]. A team that includes neurologists, physiotherapists, speech pathologists, neuropsychologists, and other specialists is essential to address the complex needs of patients, with regard to cognitive decline. Multidisciplinary care ensures that both motor and non-motor symptoms are managed, improving patients’ quality of life. This collaborative approach enables tailored interventions, early detection of cognitive changes, and more comprehensive treatment plans that support physical rehabilitation and psychological and emotional well-being [11,12].

Recent studies indicate that targeted rehabilitation, if implemented early, can slow functional decline, reduce care burden, and improve the overall quality of life for both patients and their families [11,12]. It has been demonstrated that only a synergistic and personalized approach, as well as innovative approaches, can effectively address the complexities of the disease and provide patients with significant improvements in their quality of life [13]. In this context, motor and cognitive rehabilitation has gained increasing importance, particularly with the advent of innovative technologies such as virtual reality (VR), robotics, and telerehabilitation [14,15,16,17]. Indeed, conventional rehabilitation often focuses primarily on motor skills, which can result in insufficient integration of cognitive and motor functions. This limited approach may not fully address the complexities of PD, where cognitive impairments frequently accompany motor symptoms. Innovative therapies, including VR, offer a more holistic approach by promoting motor–cognitive integration through engaging and adaptive training environments.

Recent studies have shown that VR can significantly enhance motor performance and cognitive engagement in PD patients, leading to improved rehabilitation outcomes [18,19,20]. These therapies aim to enhance both cognitive and motor functions simultaneously, thereby addressing the shortcomings of traditional methods and improving overall patient outcomes. Moreover, the integration of robotics into rehabilitation programs has been supported by research from Jiang et al. [21], which highlights the benefits of robotic-assisted training in improving functional mobility and cognitive performance in PD patients. Additionally, the growing role of telerehabilitation in extending the scope of therapeutic interventions is noteworthy. A recent work by Bianchino et al. [22] emphasizes how telerehabilitation not only provides patients with access to therapeutic resources from home but also ensures continuous monitoring and personalized feedback, further enhancing adherence and motivation. Combined with traditional physiotherapy techniques, these advanced interventions aim to improve balance, gait, and cognitive function [10,23]. In this sense, the use of VR and robotics in enhancing motor and cognitive skills may be relevant, as well as the growing role of telerehabilitation in extending the scope of therapeutic interventions [15,24].

The primary objective of this study was to evaluate the usability and patient satisfaction associated with innovative rehabilitation technologies, such as VR and robotics. We aimed to determine how well patients perceived they had achieved their individual rehabilitation goals, ensuring that the results were in line with their personalized goals. In addition, this study assessed the impact of this multidisciplinary rehabilitation pathway on cognitive function in patients with PD, specifically focusing on the often-overlooked executive functions. We also analyzed changes in psychological well-being, particularly levels of depression and anxiety. Overall, these objectives aim to provide a comprehensive assessment of the clinical benefits of a technology-based rehabilitation program and its potential to improve functional recovery and quality of life in patients with PD.

## 2. Materials and Methods

### 2.1. Study Design

This study utilized electronic medical records to collect data, ensuring accuracy and minimizing potential biases associated with scoring or interpretation. Data were retrospectively extracted for all PD patients, who completed a rehabilitation program at our institution between November 2017 and February 2020. During this period, our Institute was carrying out an experimental project, founded by the Italian Ministry of health and the Sicilian Government, through which patients with PD were admitted to a dedicated ward (20 beds) with a personalized rehabilitation project.

Although this is a retrospective study and the sample size was largely determined by the availability of patients meeting the inclusion criteria, we conducted a power analysis to ensure that the sample size would provide robust statistical results. Specifically, we aimed for a statistical power of 90% (1 − β = 0.9), a significance level of 5% (α = 0.05), and an expected moderate effect size (Cohen’s d = 0.5). The power analysis indicated that approximately 85 patients would be needed. With 104 patients included in the study, we exceeded this requirement, ensuring sufficient statistical power and reliability.

This retrospective study was conducted following the ethical principles of the 1964 Declaration of Helsinki and was approved by the Local Ethics Committee of the IRCCS Centro Neurolesi Bonino-Pulejo (PD-Innov-39/2024). Before starting the rehabilitation program, all participants provided written informed consent for the use of their data in research.

### 2.2. Patients Selection

This study included PD patients who met the following inclusion criteria: (i) diagnosis of idiopathic PD based on the Movement Disorders Society (MDS) diagnostic criteria [25]; (ii) age between 40 and 80 years; (iii) at least 5 years of formal education; and (iv) a Hoehn and Yahr disease stage below 3.

Patients were excluded if they (i) had major psychiatric disorders (e.g., major depression, psychosis); (ii) had a diagnosis of dementia according to the MDS criteria.

**Figure 1:**
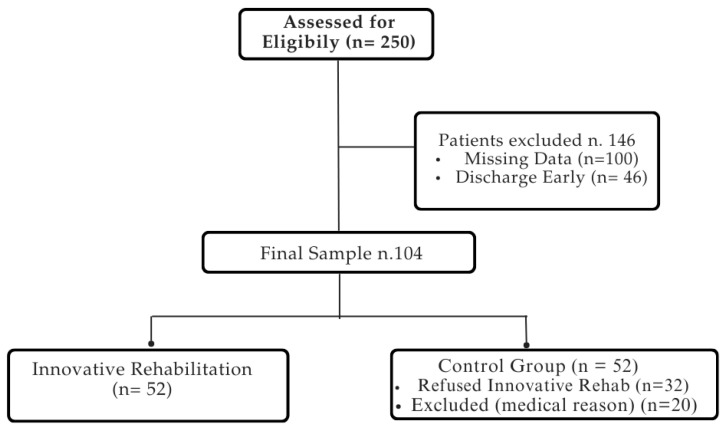
Flowchart of patient selection process.

Overall, 104 out of 250 patient records initially reviewed met the inclusion criteria and were included in the final analysis. The patient selection process is summarized in Figure 1.

### 2.3. Outcome Measures

The primary outcome measure was patient usability and satisfaction with the innovative treatments, assessed using the System Usability Scale (SUS) [26]. Additionally, the Goal Attainment Scaling (GAS) [27] was used to measure patients’ perception of goal achievement, while the Hamilton Rating Scale for Depression (HRS-D) [28] and the Hamilton Rating Scale for Anxiety (HRS-A) [29] were employed to assess psychological well-being. To reduce the risk of bias in the use of these subjective scales, several strategies were implemented. First, raters underwent specific training to ensure consistent application of the scales. Additionally, blinding was applied whenever possible, with raters unaware of the clinical status of patients and the interventions they received, minimizing the influence of expectations on outcomes. Finally, the scales were validated by comparing the scores with established external benchmarks from the literature, which helped ensure the reliability and validity of the results.

Secondary outcomes included the assessment of cognitive functions, specifically executive functions. Cognitive function was evaluated using the Mini-Mental State Examination (MMSE) [30] and the Frontal Assessment Battery (FAB) [31] to provide a comprehensive assessment of general cognitive abilities and executive functioning. The MMSE was chosen to deliver a global evaluation of cognitive functions, as it is widely validated and sensitive in detecting generalized cognitive decline, which is often present in patients with PD. Conversely, the FAB was utilized to specifically assess executive functions related to the frontal cortex, which are frequently compromised in these patients. This combined approach enables a more thorough understanding of cognitive functioning across the various domains impacted by the disease.

These scales were administered at baseline (T0) and after treatment (T1), while the SUS was administered only at the end of the treatment (T1).

### 2.4. Procedures

**Figure 2:**
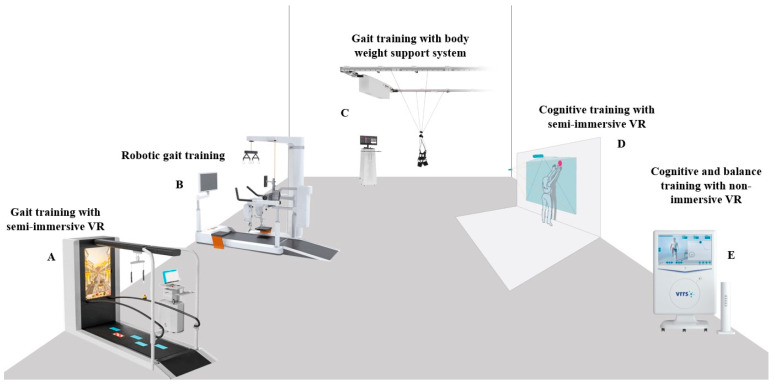
Innovative rehabilitation pathway for patients affected by PD. Legend: A = C-Mill treadmill; B = Lokomat; C = the Rysen system; D = BTS Nirvana room; E = Virtual Reality Rehabilitation System (VRRS)—Evo.

In our study, we collected data from 52 PD patients (EG) who underwent an innovative multidisciplinary rehabilitation pathway integrating advanced technologies, such as robotics, VR, and telerehabilitation (see Figure S1). These patients were compared to a control group (CG) of 52 patients who received traditional physiotherapy without the use of innovative technologies. Both groups completed the same number of rehabilitation sessions over a comparable time frame, allowing for direct comparisons between traditional and advanced multidisciplinary treatments. The comparison aimed to evaluate functional improvements to determine the effectiveness of multidisciplinary integration and cutting-edge rehabilitation technologies in the treatment of PD. Although usability and patient satisfaction were the primary outcomes of this study, we focused the analysis on cognitive function due to the completeness of the cognitive data available, which were designated as a secondary outcome. The innovative pathway incorporated both cognitive and motor rehabilitation using VR and robotics, which can also be delivered through telerehabilitation modalities. Telerehabilitation represents a significant advancement in the long-term treatment of PD, allowing patients to continue exercises at home with remote monitoring. This approach reduces the need for frequent clinic visits and helps prevent functional decline. Additionally, conventional neurorehabilitation (see Figure 2) was tailored to meet individual patient needs and capabilities, all under the supervision of a multidisciplinary team.

In this study, the CG provided a baseline comparison, reflecting the standard rehabilitation care available for PD, against which the effectiveness of the innovative multidisciplinary pathway could be assessed. Thus, the CG received only conventional physiotherapy and cognitive rehabilitation, although the same amount of treatment was provided to both groups (see Table A1 in Appendix A). Patients selected for the CG received only traditional physiotherapy that did not involve innovative technologies, such as robotic devices or VR, due to several factors. Some patients had contraindications, including musculoskeletal limitations, or personal preferences that hindered their participation in more technology-based interventions or the integration of cognitive or psychological therapy.

### 2.5. Traditional Physiotherapy

Traditional physiotherapy focused on balance and gait training exercises. These included obstacle negotiation, tandem and slalom walking, gait training, sit-to-stand exercises to improve core stability, weight-shifting exercises, and both monopodal and bipodal balance exercises. All exercises were adapted to the individual needs and capabilities of the patients, and physiotherapists closely supervised the sessions to prevent falls.

### 2.6. Innovative Rehabilitation Pathway for Patients with PD

Given the complexity of their clinical, rehabilitative, psychological, and social needs, PD patients require a personalized and multidisciplinary approach. The rehabilitation pathway started with an outpatient assessment conducted by a neurologist and a physiatrist. Patients were assigned to different rehabilitation pathways based on the severity of symptoms, measured by the H&Y scale (Table 1).

For patients with mild symptoms (H&Y 1 to 2), treatment was conducted on an outpatient basis, with two weekly sessions incorporating robotic and VR devices (CAREN, Motek, The Netherlands, Bts Nivana, BTS Bioengineering, Milano, Italy, C-Mill, Mothek, The Netherlands), according to individual needs. Subsequently, patients were monitored remotely via a telerehabilitation program. This included synchronous sessions with healthcare professionals via digital platforms specifically designed for motor and cognitive rehabilitation (e.g., the VRRS platform, Khymeia, Padova, Italia), ensuring continuity of treatment and allowing constant monitoring of progress. In case of worsening of symptoms, patients were reassessed for potential hospital admission.

For patients with moderate or severe symptoms (H&Y ≥ 2), the rehabilitation process began with hospital admission for a more intensive personalized treatment pathway. Patients were admitted to our neurorehabilitation facility for an intensive two-month program, with six days of rehabilitation per week including five to seven daily training sessions. This phase incorporated personalized rehabilitation plans based on a thorough assessment by a multidisciplinary team, including physiatrists, neurologists, physical therapists, speech therapists, psychologists, occupational therapists, and robotic nurses. The team assessed motor, cognitive, communication, and daily living skills, allowing for personalized interventions using both conventional and advanced methods such as robotics and VR.

PD patients were trained on both non-immersive and semi-immersive VR devices related to the specific motor and cognitive functions that need enhancement. Immersive VR tools, such as the CAREN system, fully envelop the user in a virtual environment, providing a highly engaging experience that simulates real-life scenarios, which can be particularly beneficial for practicing complex movements and decision-making processes in a safe setting. These immersive experiences can help improve motor skills and cognitive functions by offering patients a sense of presence and interaction with the virtual world. On the other hand, semi-immersive VR tools, like the C-Mill [32], Nirvana systems, and the Virtual Reality Rehabilitation System (VRRS), allow users to interact with a partially simulated environment. These systems provide a balance between immersion and accessibility, enabling patients to engage with tasks that challenge their motor and cognitive skills without complete immersion. The semi-immersive setup allows for some physical interaction while still maintaining elements of the real world, making it suitable for patients who may require a gentler introduction to VR technology. Lastly, non-immersive VR devices present exercises on standard screens, allowing patients to engage with VR-based activities in a more traditional way. While these devices lack the immersive qualities of their counterparts, they still provide valuable training opportunities that can be tailored to individual patient needs and capabilities. By incorporating a range of VR tools, immersive, semi-immersive, and non-immersive, we aim to create a comprehensive rehabilitation program that caters to the diverse needs of PD patients, enhancing both their motor and cognitive recovery.

The physiatrist assessed the patient’s general functional status, while neurologists assessed sensory and motor deficits. The physical therapists used a combination of subjective scales and objective tools, including motion capture systems, assessing gait and posture. Speech–language pathologists focused on communication challenges, while occupational therapists addressed activities of daily living. Neuropsychologists assessed cognitive and emotional dimensions, guiding psychotherapeutic and cognitive interventions.

The resulting Individual Rehabilitation Plan (IRP) followed the International Classification of Functioning, Disability, and Health (ICF) model, incorporating therapies designed to meet the patients’ motor and cognitive rehabilitation needs. Using innovative devices, there was improved motivation and treatment accuracy, with continuous monitoring and adjustments based on progress. This ensured adaptive rehabilitation aimed at improving the patient’s functional outcomes.

After the two-month hospital stay, patients moved to a day hospital (DH) program, attending three times a week for further intensive rehabilitation. DH sessions combined conventional therapies, VR-based interventions, and robotic treatments, customized to individual cognitive and motor needs. This phase ensured continuity of care and further promoted recovery.

After the DH program, patients continued their rehabilitation at home via telerehabilitation, ensuring sustained progress and preventing functional decline.

This comprehensive pathway responded to the changing needs of PD patients, improving outcomes and potentially their quality of life, as demonstrated in previous studies on patients with multiple sclerosis and spinal cord injury [33,34].

### 2.7. Statistical Analysis

The data were analyzed using Jamovi version 2.3 (Jamovi Foundation), considering *p* < 0.05 as statistically significant. Descriptive statistics were reported as mean ± standard deviation or median ± first-third quartile for continuous variables, as appropriate. Frequencies (%) were used for categorical variables. Given that the data subjected to the Shapiro–Wilk test were presented with a normal distribution, a post hoc *t*-test for group differences in time and performance was conducted using Student’s *t*-tests, with the Bonferroni correction (0.00125). A paired *t*-test was utilized in the post hoc analysis to compare cognitive performance at T0 and T1.

## 3. Results

The medical records of 250 patients with PD were included in the analysis utilizing electronic recovery system data. The final sample consisted of 104 patients, all of whom completed the rehabilitation process without reporting any side effects. The demographic and clinical characteristics of the sample are reported in Table 2.

No significant statistical differences were detected between the two groups at baseline in terms of age (*p* = 0.07), gender (*p* = 0.86), and education (*p* = 0.41). Additionally, no significant differences were observed in the scores of the tests/scales at T0: the MMSE (*p* = 0.08), FAB (*p* = 0.25), HRS-D (*p* = 0.68), HRS-A (*p* = 0.32), and GAS (*p* = 0.07). However, after the rehabilitation training, significant differences were identified between the groups regarding these scales (*p* < 0.001), indicating distinct effects of the training.

In the EG, we observed a statistically significant improvement in all tests/scales considered (*p* < 0.001). In the CG, patients showed significant improvement in GAS (*p* < 0.001), with patients selecting improvement in balance and walking as their primary objectives. However, while there was an improvement in executive functions (FAB *p* = 0.003) and in mood, as measured by the HRS-D (*p* = 0.0012), this enhancement was notably more limited compared to the improvements observed in the EG. No statistically significant results were found for the other scales: MMSE (*p* = 0.23) and HRS-A (*p* = 0.12). For more details, see Table 3.

## 4. Discussion

PD is a multifaceted neurodegenerative condition that requires a comprehensive and individualized approach, as highlighted in this study.

### 4.1. Usability and Patient Satisfaction

This study aimed to assess the usability and acceptability of personalized rehabilitation strategies in PD patients, integrating advanced technologies such as robotics and VR, while exploring their impact on cognitive and emotional functions. The multidisciplinary nature of this care reflects the need for a broad-based team of professionals (neurologists, physiatrists, physical therapists, speech therapists, neuropsychologists, and occupational therapists), each targeting specific domains affected by PD [10,35]. Our results demonstrate that PD patients responded positively to personalized rehabilitation strategies incorporating advanced technologies like VR and robotics, with high acceptability and usability ratings. These interventions were effective in maintaining patient engagement, a key factor for long-term treatment adherence. Recent findings aligned with our results, indicating that technology-based rehabilitation, when personalized, improved the user experience and enhanced therapy adherence. A study by Maier et al. [36] showed that VR-based rehabilitation systems specifically made to help patients foster higher patient satisfaction, leading to more consistent participation in rehabilitation sessions. Some studies highlighted that customized rehabilitation plans, which are adaptable and responsive to individual progression, foster greater motivation, and improve patient outcomes [33,34,36,37]. This adaptability is essential, especially in addressing motor and cognitive deficits in PD patients, whose symptoms can fluctuate daily.

The key factors enhancing usability were simple controls, clear instructions, and features designed to meet patient needs. In our cohort, these aspects played a crucial role in patient participation and engagement. Additionally, interventions like VR and robotics that adjust task complexity and provide real-time feedback boost patient engagement through high usability scores, as demonstrated in usability studies of neurorehabilitation devices [14,15,17]. The seamless integration of these technologies into personalized rehabilitation plans provided patients with greater comfort and confidence, facilitating both cognitive and motor improvements [33,34]. Moreover, patient-centered approaches, where the system adapts dynamically based on user interaction and progression, have consistently been shown to increase therapeutic success. Moreover, robotics and VR-based therapies furnished a significant portion of our treatment protocol, particularly for patients with more advanced stages of PD (H&Y ≥ 2), providing novel avenues for targeting motor and cognitive recovery. In recent studies, VR systems and robotic devices have shown great potential in terms of patient acceptability, especially when treatments are tailored to individual needs [33,37,38]. Finally, our results are consistent with those of Leventhal et al. [37], who also demonstrated that patient-centered, technology-enhanced interventions result in better compliance and satisfaction, which is critical for long-term rehabilitation programs, leading to greater patient satisfaction and perceived effectiveness [39].

### 4.2. Cognitive Function

The secondary objective of this retrospective study was to assess the impact of the innovative multidisciplinary rehabilitation pathway on cognitive function. In our study, the results of the MMSE and FAB in the control group showed less significant improvements compared to the experimental group, suggesting that the technological and personalized approach of VR may be more effective in promoting cognitive enhancements in executive functions, which are often neglected in standard PD rehabilitation. This focus is critical, as cognitive impairment, especially in executive functions, is prevalent in PD and often inadequately addressed in conventional rehabilitation settings. Executive dysfunction, encompassing difficulties in planning, decision-making, and inhibitory control, profoundly impacts daily living and quality of life [39,40,41]. The use of VR-based cognitive training directly and appropriately addressed these deficits, leading to significant cognitive improvements (with regard to executive function) and filling a notable gap in current therapeutic approaches. After the conventional cognitive training, indeed, the CG had a less pronounced enhancement in cognitive measures, as per the MMSE and FAB.

We hypothesize that the enhanced improvement in cognitive abilities among PD patients undergoing VR therapy is closely linked to the neuroplasticity induced by these immersive environments, which could foster neural adaptations and improve cognitive functions, as demonstrated by various studies [15,17,39,41]. This neuroplasticity is facilitated by the activation of specific neural circuits involved in executive functions, particularly in the prefrontal cortex, which is crucial for higher-order cognitive processes. Additionally, VR therapy supports motor learning through the engagement of the mirror neuron system, which is essential for understanding and imitating movements. This system allows patients to observe virtual actions and simulate them in their minds, improving cognitive and motor skills. The immediate feedback provided by VR systems could facilitate the knowledge of results, leading to a better understanding of their progress. Moreover, the interactive and engaging aspects of VR promote reinforcement learning, enabling repeated practice of cognitive and motor tasks in a structured and enjoyable format. This repetition can lead to long-term potentiation, a neurophysiological process associated with strengthened synaptic connections. Together, these factors contribute to a more effective rehabilitation experience, leading to significant cognitive and motor improvements in our PD patient cohort.

### 4.3. Psychological Well-Being

In addition to cognitive improvements, psychological well-being was another crucial outcome in our study. Monitoring depression and anxiety levels using the HRS-D and HRS-A provides insights into the mental health status of patients, which is crucial for ensuring optimal recovery. The significant improvements observed in the EG reflect not only cognitive gains but also enhancements in overall psychological well-being, suggesting a synergistic effect of addressing both cognitive and emotional aspects of rehabilitation. This is consistent with previous findings, highlighting the connection between cognitive function and emotional health in PD patients [42]. The usability of the technology likely contributed to these improvements, as patients were more comfortable and engaged in the rehabilitation process.

### 4.4. Telerehabilitation Effects

One of the most innovative aspects of our rehabilitation pathway was the integration of telerehabilitation, which allowed for the continuation of care following both outpatient and inpatient interventions [24]. Telerehabilitation in PD is increasingly recognized as a viable solution for providing consistent and high-quality care while reducing the burden of hospital visits [14,43,44,45,46]. In our study, telerehabilitation at home involved advanced technologies to facilitate remote therapy sessions for patients with PD. This approach allowed patients to engage in rehabilitation exercises from the comfort of their homes while receiving support and guidance from a multidisciplinary team. Patients were equipped with wearable devices and digital platforms to track their progress and monitor vital signs in real-time. These data enabled healthcare professionals to assess the effectiveness of the rehabilitation program and make necessary adjustments. The telerehabilitation program utilized mainly VR systems to create semi-immersive rehabilitation experiences, allowing patients to participate in interactive exercises designed to enhance cognitive and motor functions, tailored to their individual needs. Regular video calls were scheduled between patients and their rehabilitation team, providing opportunities for therapists to offer real-time feedback, modify exercises, and ensure that patients maintained proper form and motivation. Each patient’s rehabilitation program was personalized based on their specific goals, abilities, and progress, enhancing engagement and adherence to the process. Telerehabilitation reduced the need for frequent in-person visits, making rehabilitation more accessible for patients who may have mobility challenges or live far from healthcare facilities. This approach aimed to prevent functional decline by ensuring continuous engagement in therapy. By implementing telerehabilitation, we aimed to improve patient outcomes and satisfaction while addressing the challenges posed by traditional rehabilitation methods in the context of PD. Our findings suggested that telerehabilitation might not only help maintain functional gains post-discharge but also prevent the functional decline often seen when intensive rehabilitation is discontinued. Recent studies demonstrated the feasibility and effectiveness of telerehabilitation in PD management. For instance, a randomized controlled trial by Isernia et al. found that telerehabilitation was as effective as in-person visits for managing motor and non-motor symptoms in PD patients [47]. Furthermore, other studies argued that telemedicine, combined with VR or robotic interventions, could be the future of PD rehabilitation, particularly in the context of personalized care [17,39,41].

## 5. Study Limitations and Future Direction

While this study offers promising insights into PD rehabilitation, several limitations need to be acknowledged. First, the focus of our study was predominantly on usability and cognitive and psychological outcomes, and we did not specifically assess motor outcomes, although they are a central aspect of PD. While motor deficits are a hallmark of PD and are often targeted in rehabilitation, the decision to exclude motor assessments was based on the absence of complete motor data for many subjects. Future studies should integrate motor outcome measures to provide a more comprehensive picture. Second, this study did not include long-term follow-up, which is critical in determining the sustainability of rehabilitation gains. Additionally, the retrospective nature of this study limits the ability to establish causal relationships, and larger prospective randomized clinical trials are needed to confirm these findings and explore long-term effects. Finally, the generalizability of the findings may be limited by the relatively small sample size and specific study population.

Since cognitive and motor deficits are interconnected in PD, understanding the dual impact of rehabilitation on these domains could improve the development of personalized treatment plans. Furthermore, incorporating larger multicenter prospective randomized clinical trials will be critical to confirm the efficacy of the innovative technologies used in this study, such as virtual reality and robotics, and to evaluate their long-term benefits. Future studies should also explore the potential role of neuroimaging and biomarkers to better understand the neurophysiological changes associated with rehabilitation. Finally, extending the scope to examine the effects of rehabilitation on other quality-of-life indicators, such as social functioning, could provide a more holistic view of patient recovery and further personalize rehabilitation strategies to meet the diverse needs of PD patients.

## 6. Conclusions

In conclusion, this study supported the notion that a personalized, technology-based rehabilitation program could significantly enhance cognitive function and psychological well-being in patients with PD. The innovative multidisciplinary pathway employed in this study provides a valuable model for future rehabilitation programs, emphasizing the need for comprehensive, tailored approaches that address the multifaceted needs of PD patients. Further research is warranted to explore long-term effects and to optimize these interventions, ensuring that the benefits observed in this study can be sustained over time and translated into improved quality of life for patients living with PD.

## Figures and Tables

**Table 1:** Phases of the rehabilitation pathway for Parkinson’s disease patients.

Phase	Setting	Description	Frequency/Duration
Mild Cases (H&Y 1–2)			
1. Initial Assessment	Outpatient	Neurologist and physiatrist evaluate the severity of symptoms using the H&Y scale.	1 day
2. Outpatient Rehabilitation	Outpatient	Personalized rehabilitation using robotic devices and VR for motor/cognitive functions based on patient needs.	2 sessions per week (until re-evaluation or symptom worsening)
3. Tele rehabilitation	Home	Patients are monitored remotely through synchronous telerehabilitation using the VRRS platform.	Continuous, with regular re-evaluations
Moderate-to-Severe Cases (H&Y ≥ 2)			
1. Initial Assessment	Outpatient	Neurologist and physiatrist evaluate the severity of symptoms using the H&Y scale.	1 day
2. Intensive Hospitalization	Inpatient (Hospital)	Patients undergo intensive rehabilitation (6 days/week, 5–7 training sessions/day) with personalized interventions.	2 months
3. Day Hospital (DH)	Day Hospital	Post-hospitalization, continued rehabilitation through conventional, robotic, and VR-based therapies. Some moderate cases receive rehab only in DH.	3 sessions per week for 6–8 weeks
4. Tele rehabilitation Follow-up	Home	Continued home-based rehabilitation through telerehabilitation to maintain progress.	Continuous (following DH completion)

**Table 2:** Demographic and clinical characteristics of the patients.

	All Patients	Experimental Group	Control Group	*p*-Value
Patients number	104	52	52	
Age (years)	63.3 ± 9.32	61.6 ± 10.3	65.0 ± 8.0	0.07
Gender				0.86
Female	47 (45.2%)	24 (46.15%)	23 (44.23%)	
Male	57 (54.8%)	28 (53.85%)	29 (55.77%)	
Education	11.3 ± 3.59	11.0 ± 3.56	11.6 ± 3.62	0.41
Disease Duration	7 ± 2	7.1 ± 1.9	6.9 ± 2.1	0.87

Mean ± standard deviation was used to describe continuous variables; proportions (numbers and percents) were used to describe categorical variables.

**Table 3:** Post hoc analysis of clinical scores between baseline (T0) and follow-up (T1), for both experimental (EG) and control (CG) groups.

Clinical Assessment	Experimental Group		*p*-Value	Control Group		*p*-Value
	T0	T1		T0	T1	
MMSE	**27.1 (±2.03)**	28.4 (±1.35)	<0.001 **	24.8 (±3.59)	25.3 (±3.32)	0.23
FAB	15.2 (±2.95)	16.6 (±2.34)	<0.001 **	13.6 (±2.53)	14.1 (±2.47)	0.003 *
HRS-D	14.2 (±8.06)	11.7 (±7.79)	<0.001 **	14.8 (±7.30)	13.4 (±5.80)	0.012 *
HRS-A	15.3 (±7.60)	12.9 (±7.11)	<0.001 **	16.8 (±8.00)	15.2 (±7.55)	0.12
GAS	37.8 (±1.41)	63.6 (±5.04)	<0.001 **	37.4 (±0.491)	46.9 (±8.13)	<0.001 **
SUS	81.0 (±8.98)			57.0 (±11.1)		

Scores are shown as average (±standard deviation); significant differences are in bold. Legend: Frontal Assessment Battery (FAB); Goal Attainment Scaling (GAS); Hamilton Rating Scale for Depression (HRS-D); Hamilton Rating Scale for Anxiety (HRS-A). Mini-Mental State Examination (MMSE); System Usability Scale (SUS). Bonferroni correction *p* < 0.0125. Signif. codes: ** *p* < 0.001; * *p* < 0.01.

## Data Availability

The original contributions presented in the study are included in the article/Supplementary Material, further inquiries can be directed to the corresponding author.

## Supplementary Materials

The following supporting information can be downloaded at https://www.mdpi.com/article/10.3390/biomedicines12112426/s1, Figure S1: Innovative pathway.

## Appendix A

**Table A1:** Description of the devices.

Motor Domain	Timing	EG VR/Robotic Rehab Plus Conventional Training	Setting or Device	CG Conventional Training	Setting
	**Non-immersive VR and/or BWS system**				
**Balance**	EG: 60 min of robotic or VR rehab 60 min of conventional physiotherapy CG: 120 min of conventional physiotherapy	Patients with low or moderate disease severity performed balance training with VRRS Evo (e.g., weight-shifting exercise in which the subject has to collect objects, like balls, with different sizes and colors) plus conventional physiotherapy (see description below). Patients with moderate balance impairment received training sessions with the Rysen system to train static balance (e.g., standing with a decreased base of support or on an unstable support surface, controlling heel–toe imbalances, and shifting the center of gravity) and dynamic balance (e.g., walking with frequent stops and changes in direction, and postural variations).	VRRS: non-immersive VR system to train motor, cognitive, and postural functions. The Rysen system (Motek, The Netherlands) is a 3D BWS system designed to promote the functional recovery of walking by enhancing balance reactions. The safety system consists of a harness fixed to a swing bar by means of four weight lock buckles, which protect the patient from falls.	Conventional physiotherapy for static balance included: standing with different variations in the base of support, tandem standing, and shifting the center of gravity using an oscillating platform. Dynamic balance activities included: standing up and sitting down from a chair with or without using hands, walking in tandem, lateral weight shifting, stationary stepping, walking with frequent stops and changes in direction, and postural variations.	Traditional rehabilitation gym
**Semi-immersive VR and/or robotic gait training with VR screen**					
**Gait**	EG: 60 min of robotic/VR rehabilitation + 60 min of conventional physiotherapy CG: 120 min of conventional physiotherapy	Patients with low or moderate disease severity received VR gait rehabilitation through C-Mill. Patients on C-Mill performed gait with virtual obstacles projected on the treadmill, with audio-visual stimuli according to target. Gait in tandem with virtual traffic cones, or virtual country paths projected on the treadmill, in addition to audio-visual stimuli. Walking on treadmill at different speeds based on virtual exercises (e.g., “Italian Alps”), projected on frontal screen. Walking on treadmill with frequent lateral direction changes according to virtual projections on the screen (e.g., Arkanoid). In severe stages of the disease, patients performed robotic rehabilitation through Lokomat.	The C-Mill system is an advanced treadmill with a semi-immersive VR setup, featuring body weight sensors, a harness, and integrated audio-visual stimuli. PD patients train on the treadmill while interacting with a large VR screen. The Lokomat is an exoskeleton with actuators at the hip and knee joints, providing body weight support and treadmill-based gait training. The system adapts to individual needs, initially offering 100% guidance force and 80% body weight support, which is adjusted as the patient’s abilities improve.	Gait with obstacles (e.g., bricks, boxes) with different shapes and colors. Gait in tandem with paths created by physiotherapists, using chairs, boxes, traffic cones, and sandbags. Walking at different speeds according to audio stimuli provided by therapist (e.g., music and voice of therapist). Walking with frequent direction changes according to audio (e.g., music and voice of therapist) and visual (e.g., colored tape on the floor) stimuli.	Traditional rehabilitation gym
**Cognitive and Behavioral** **Domain**	**Timing**	**EG** **VR Rehabilitation plus Conventional Training**	**Setting or Device**	**CG** **Standard Cognitive** **Training**	
		**Non-immersive VR device**			
Orientation Attention Processes Memory Abilities Executive Functions Ideomotor Constructive Praxia	EG: 45 min VR rehabilitation + 45 min standard cognitive training CG: 45 min standard cognitive training	Patients with low or moderate disease severity performed cognitive training with VRRS Evo, e.g., to stimulate verbal memory using mnemonic techniques and strategic skills through a pc-based task and virtual exercises such as trying to recall the words of a song or a written text, or poetry after reading, using VRRS and pc-verbal based auditory task; to promote the recovery of executive functioning, the cognitive therapist asks to the patient to realize specific activities to stimulate categorization skills and semantic and phonemic categorization; activities planning and logical association; tasks of analogical reasoning using a pc-based approach and virtual 2D and 3D activities plus standard cognitive training.	VRRS: non-immersive VR system to train motor, cognitive, and postural functions, characterized by an augmented sensory feedback and a human–web setting interface. Virtual interaction mediated by VRRS tool included 3 levels of difficulty for execution’s time and the numbers of stimuli-target and distractors administered.	Paper-and-pencil task-oriented stimulation involves face-to-face rehabilitation between the patient and therapist, using exercises of varying complexity to enhance cognitive abilities. Activities include memory recall, categorization, planning, and reasoning tasks, all performed with traditional paper-and-pencil tools.	
