# Supplementary figures and images for: Advances in the Neuro-Rehabilitation of Parkinson’s Disease: Insights from a Personalized Multidisciplinary Innovative Pathway
Source: Biomedicines. 2024 Oct 23;12(11):2426. doi: 10.3390/biomedicines12112426 (PMC11591689; doi:10.3390/biomedicines12112426)

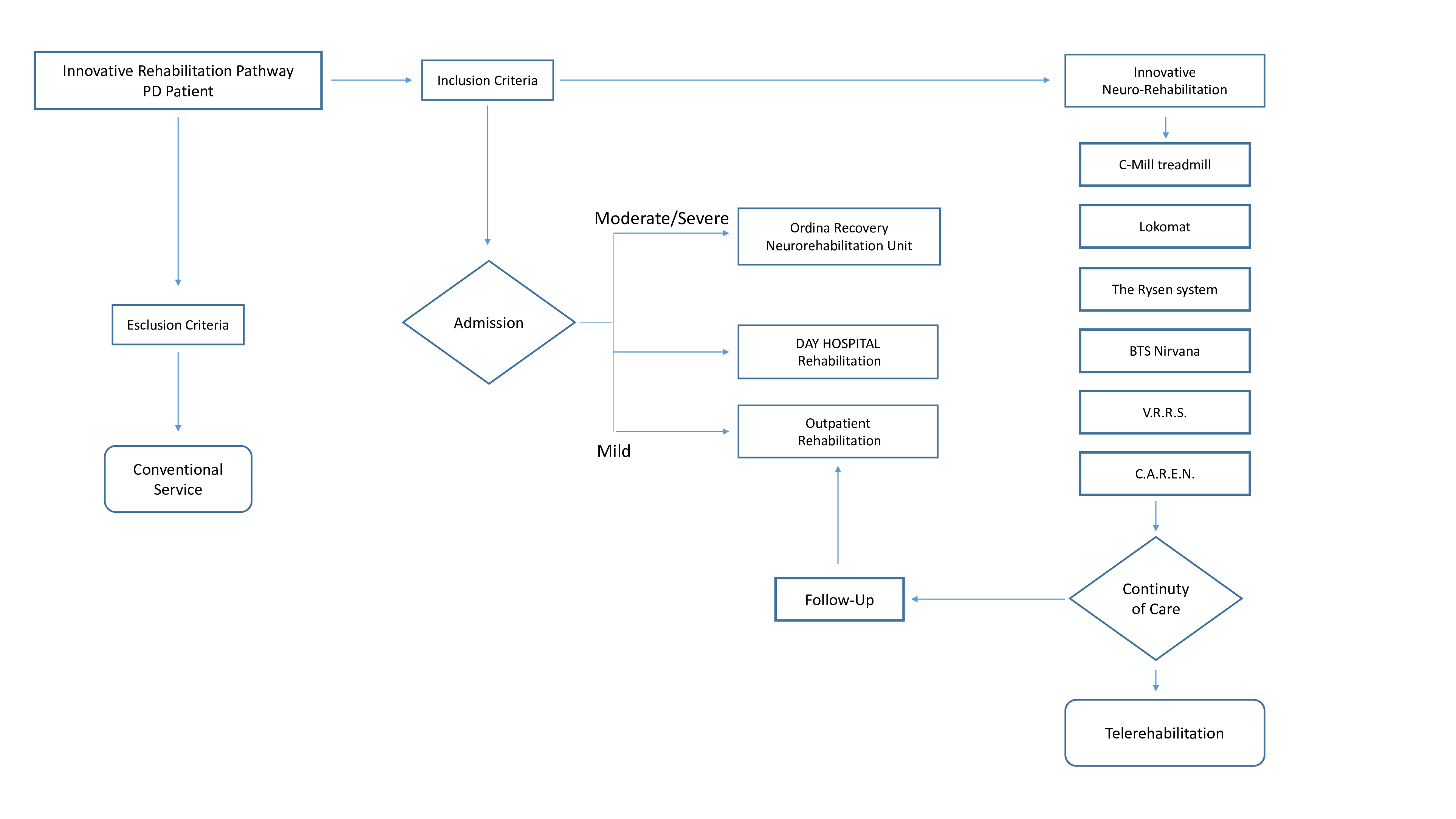

Supplement: Supplementary file 1 [file biomedicines-12-02426-s001.zip › biomedicines-3264435-supplementary.png]
